# Initial Experience of Single-Port Robotic Lobectomy for Large-Sized Non-Small Cell Lung Cancer: A Single-Center Retrospective Study

**DOI:** 10.3390/cancers16173091

**Published:** 2024-09-05

**Authors:** Jun Hee Lee, Byung Mo Gu, Hwan Seok Yong, Soon Young Hwang, Hyun Koo Kim

**Affiliations:** 1 Department of Thoracic and Cardiovascular Surgery, Guro Hospital, Korea University College of Medicine, Seoul 08308, Republic of Korea; lee2632@naver.com (J.H.L.); luvotomy7@naver.com (B.M.G.); 2 Department of Radiology, Guro Hospital, Korea University College of Medicine, Seoul 08308, Republic of Korea; yhwanseok@naver.com; 3 Department of Biostatistics, Korea University College of Medicine, Seoul 08308, Republic of Korea; hwangsy214@gmail.com

**Keywords:** robotics, robotic-assisted thoracic surgery, single-port, uniportal, video-assisted thoracic surgery

## Abstract

**Simple Summary:**

Robotic-assisted thoracic surgery (RATS) has gained popularity worldwide; however, its use in large-sized non-small cell lung cancer (NSCLC) is controversial. Our study evaluates the feasibility of single-port RATS lobectomy in patients with large-sized NSCLC (larger than 5 cm) and compares its perioperative outcomes with those of two-port RATS lobectomy. This study demonstrated that single-port RATS is comparable to two-port RATS for large-sized NSCLC. Our findings suggest that single-port RATS is feasible and can be an alternative surgical option for large-sized tumors.

**Abstract:**

Single-port robotic-assisted thoracic surgery (SP-RATS) lobectomy using the da Vinci Xi system has been performed by several pioneers. However, due to the severe collisions and the steep learning curve, this approach is not yet widely used. This study aimed to evaluate the feasibility of SP-RATS lobectomy for large-sized non-small cell lung cancer (NSCLC). As we believe that for large-sized tumors it is reasonable to make a slightly larger incision, we performed SP-RATS lobectomy for large-sized NSCLC (greater than 5 cm) through a single incision (6–8 cm). Eleven patients underwent SP-RATS lobectomy using the da Vinci Xi system at our institution from April 2022 to May 2024. The median tumor size on computed tomography and on pathology was 6.6 cm [interquartile range (IQR), 6.1–7.5 cm] and 6 cm [IQR, 5.1–7.1], respectively. The median total operative time was 198 min [IQR, 159–260 min], and the median postoperative length of stay was 4 days [IQR, 4–10 days], with no major postoperative complications (≥grade III on the Clavien–Dindo classification). Our approach may combine the benefits of single-port surgery with those of robotic surgery and is safe, feasible, and may promote better outcomes in patients with large-sized NSCLC.

## 1. Introduction

Lung cancer is the leading cause of cancer-related death, and non-small cell lung cancer (NSCLC) accounts for 85% of these cases [1,2]. Lobectomy is considered the standard approach for patients with early NSCLC [3], whereas the optimal treatment strategy for large-sized NSCLC is still unclear. Tumor size is a powerful predictor of prognosis in patients with NSCLC [4]. According to the eighth edition of the TNM stage classification for lung cancer, a tumor size of 5 to 7 cm is classified as T3, and a tumor size of 7 cm or larger is classified as T4 [5]. Moreover, for patients with a large-sized (>5 cm) NSCLC (T3, T4) without lymph node involvement and eligible for resection, lobectomy is preferred [3]. Previously, large-sized tumors were considered a contraindication for minimally invasive thoracic surgery. Although debate continues regarding the size threshold beyond which minimally invasive thoracic surgery is not recommended, many experienced surgeons argue that large tumor size is not a contraindication for a minimally invasive thoracic surgery approach. Few studies have shown that robotic-assisted thoracic surgery (RATS) is feasible even in patients with large-sized tumors [6,7]. Large-sized tumors make it challenging to obtain an adequate surgical view and often invade the bronchus, blood vessels, and other structures, making radical resection technically demanding. However, experienced robotic surgeons can facilitate these complex surgeries using the robotic system.

Since Melfi et al. initially reported RATS lobectomy in 2002 [8], it has gained popularity due to its advantages, including tremor filtration, better ergonomics, enhanced 3D view, and excellent maneuverability [9]. The conventional RATS technique utilizes two to five ports with or without a utility incision [10,11,12,13,14]. Recently, some pioneers have reported performing three-arm single-port RATS (SP-RATS) lobectomy using the da Vinci Xi robotic surgical system (Xi) (Intuitive Surgical Inc., Sunnyvale, CA, USA) [15]. However, Xi was not developed for single-port (SP) surgery; therefore, this technique has several limitations, including the collision of instruments and a steep learning curve for both the surgeon and the assistant.

We were the first in the world to report perioperative outcomes of the three-arm, two-port RATS lobectomy using a 3–4 cm utility incision with a 12 mm port for patients with NSCLC [12]. In our center, the two-port RATS approach was preferred for large-sized NSCLC. However, in some cases, after the procedure, we enlarged the utility incision to remove large-sized tumors; in cases where we did not enlarge the incision, the specimen was sometimes severely ruptured during removal. As we believe that for large-sized tumors it is reasonable to make a slightly larger incision, we decided to perform a three-arm SP-RATS lobectomy using a 6–8 cm utility incision for large-sized NSCLC. A utility incision size range of 6–8 cm is sufficient to simultaneously insert three arms through a single incision with minimal collisions. This approach is technically less demanding than the conventional SP-RATS technique using a 3–4 cm utility incision. Potential benefits of this approach are less pain, avoiding rib spreading without resection, rapid docking and undocking, and easier conversion to video-assisted thoracic surgery (VATS). However, there have been no studies evaluating this technique. Therefore, the aim of this study was to report the surgical details of the SP-RATS approach and evaluate its safety and feasibility.

## 2. Materials and Methods

### 2.1. Study Design and Patient Selection

We retrospectively collected data from patients who underwent RATS lobectomy using the Xi and VATS lobectomy for large-sized NSCLC at the Korea University Guro Hospital from March 2019 to May 2024. Large-sized NSCLC is defined as a solid tumor, larger than 5 cm, on a preoperative chest computed tomography (CT) scan. All procedures were performed by a single surgeon (H.K.K). The patients were divided into three groups: the VATS group, the two-port RATS (TP-RATS) group, and the SP-RATS group. We began with a 3-arm TP-RATS approach for large-sized NSCLC in March 2019 and converted to a 3-arm SP-RATS approach in April 2022.

The indications for the SP-RATS approach are similar to those of the conventional RATS approach [12,16]. The contraindications include radiotherapy, fibrothorax, and central vascular structure invasion. Previous chemotherapy and chest wall invasion were not considered contraindications.

### 2.2. Perioperative Data

Preoperative chest CT was performed within one month prior to surgery. Tumor size was estimated by chest CT and was deemed to be the largest measurement on the three-diameter assessment made by an experienced thoracic radiologist. All diameters were measured in 1 mm increments. Tumor size on pathology was obtained from the pathology report, and the measurements were in units of 1 mm. Pathologic staging was assessed according to the 8th edition of the TNM staging manual for lung cancer [5]. Postoperative pain was assessed three times per day using a visual analog scale (VAS). Postoperative complications were monitored for a period of up to 30 days and assessed according to the Clavien–Dindo classification [17]. Minor complications were categorized as Grade I–II, while major complications were categorized as Grade III–V.

### 2.3. Operative Techniques

**Figure 1:**
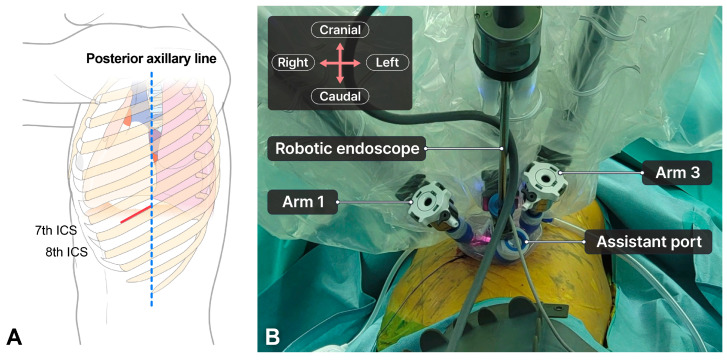
Port mapping for single-port robotic-assisted thoracic surgery for large-sized tumors. (**A**) The anatomy for an incision site. The red line indicates an incision site. The blue dotted line indicates the posterior axillary line. A 6–8 cm utility incision is made at the 7th–8th ICS. (**B**) Layout of robotic arm positions. During the procedure, an 8 mm port is used on both arms (arm 1 and arm 3) to reduce collisions (Figure 2A). When using the robotic stapler, the 8 mm port on either arm 1 or arm 3, where the stapler will be inserted, is replaced with a 12 mm port (Figure 2B). After stapling, we replaced the 12 mm port with an 8 mm port to reduce any collisions between instruments.

The patient was placed in the lateral decubitus position with one-lung ventilation. For the SP-RATS approach, a 6–8 cm incision was made between the mid-axillary line and the posterior axillary line at the 7th–8th intercostal space (ICS), depending on the tumor location (Figure 1A). A wound retractor was inserted through the incision, and a multichannel port (LapSingle Vision; Sejong Medical, Paju, Republic of Korea) was installed. We insufflated carbon dioxide (CO_2_) gas at a pressure of 6–10 mmHg during the procedure. The patient cart was placed on the left side of the patient. The robotic scope was placed in the middle of the incision, with two 8 mm ports placed at the left and right edge of the incision (Figure 1B). We preferred using Cadiere forceps on the left arm (arm 1), the robot endoscope on the middle arm (arm 2), and Maryland bipolar forceps on the right arm (arm 3). Arm 4 was usually inactive and moved out. The space between the robotic arms was sufficient for the assistant to insert instruments, which enhanced the surgical view, lung traction, and suction.

To further minimize collisions, the robotic arm and the robot endoscope moved incrementally and worked in parallel. Although usual VATS instruments have four degrees of freedom (pitch, yaw, rotation, and in-out), the robotic arm in the da Vinci Xi system has seven degrees of freedom (wristed pitch, wristed yaw, grasp, yaw, pitch, rotation, and in-out) [18]. In our approach, yaw motion is limited, but other movements are not restricted (Video S1). This allows meticulous surgery to be performed in narrow spaces, compared to VATS. The surgical steps were similar to those of conventional RATS (Video S2). Due to the absence of tactile sense in the robotic system, a vessel loop was used for the retraction of the vessels and bronchus. Additionally, placing rolled gauze between the robotic wrist and structure facilitated traction [12].

The resected specimen was removed using the retrieval bag (Lapbag; Sejong Medical, Paju, South Korea) through an incision without a rib spreader. A complete mediastinal lymph node dissection was performed, and a chest tube (20 Fr or 24 Fr) was inserted at the anterior edge of the incision. The incision site was at a lower ICS level (7th–8th), but because it was wide, conversion to VATS was not difficult and did not require an additional incision. If conversion to two-port approach was necessary, a 12 mm port was made at the 6th–7th ICS level on the anterior axillary line. If conversion to thoracotomy was necessary and the incision did not provide an adequate surgical view due to its low level, a new incision was made at the 5th ICS level.

For the TP-RATS approach, as described in a previous report [12], a 3–4 cm utility incision was made at the 7th–8th ICS level in the posterior axillary line with a 12 mm port at the 6th–7th ICS level in the anterior axillary line. The surgical steps were similar to those of the SP-RATS approach. When the specimen could not be removed through the incision, we enlarged the incision size. A complete mediastinal lymph node dissection was performed, and a chest tube (20 Fr or 24 Fr) was inserted through the 12 mm port. The postoperative management was the same for both groups, as described in a previous publication. If conversion to three ports was necessary, a 12 mm port was made at the 8th–9th ICS level along the scapular line, with an 8 cm distance between the utility incision and the 12 mm port.

For the VATS approach, as described in a previous report [19], a 2.5–4 cm utility incision was made at the 5th ICS level. In our center, the standard VATS approach is SP-VATS. Complete mediastinal lymph node dissection is routinely performed.

### 2.4. Statistical Analyses

SPSS version 27 (IBM Inc., Armonk, NY, USA) was used for statistical analysis. Continuous variables are expressed as median and interquartile range (IQR). The Mann–Whitney U test was used to compare continuous variables. Categorical variables are expressed as numbers and percentages. For categorical variables, Pearson’s chi-square test and Fisher’s exact tests were performed as needed. Statistical significance was considered at *p* < 0.05.

### 2.5. Ethical Statement

This study was approved by the Institutional Review Board (2024GR0310). The requirement to obtain informed consent was waived owing to the retrospective study design and its minimal risk. This study was conducted in accordance with the principles of the Declaration of Helsinki (revised in 2013).

## 3. Results

A total of 64 patients were included in this study, with 11 patients in the SP-RATS group, 24 patients in the TP-RATS group, and 29 patients in the VATS group.

### 3.1. Clinical Characteristics of Patients Who Underwent SP-RATS

Table 1 shows the detailed characteristics of the 11 patients who underwent the SP-RATS lobectomy for large-sized NSCLC. Median incision size was 7 cm [IQR, 6–8 cm]. All patients were discharged within 15 days after surgery and without major postoperative complications. There were no technical operative issues associated with the maneuvers of the robotic arms. Robotic arm movements (seven degrees of freedom) can be performed appropriately with minimal collisions (an exception was a patient with a narrow ICS, who required an additional port; thus, there was one case of conversion to the two-port approach).

### 3.2. Comparative Analysis between the TP-RATS Approach and VATS Approach

Table 2 presents the inter-group comparative analysis of patient characteristics. The median tumor sizes on preoperative chest CT were 6.6 cm [IQR, 6.1–7.5 cm], 6.2 cm [IQR, 6.0–7.0 cm], and 6.2 cm [IQR, 5.0–7.5 cm] for SP-RATS, TP-RATS, and VATS, respectively (*p* > 0.05). Similarly, the median tumor sizes on pathology were 6.0 cm [IQR, 5.1–7.1 cm], 5.5 cm [IQR, 5.1–6.0 cm], and 6.0 cm [IQR, 5.3–7.7 cm] for SP-RATS, TP-RATS, and VATS, respectively (*p* > 0.05). There were no significant differences between the three groups (*p* > 0.05).

Table 3 provides a summary of the perioperative outcomes in the three groups. The median total operative time was 198 min [IQR, 159–260 min], 181.5 min [IQR, 165.5–212.5 min], and 178 min [IQR, 154–232 min], respectively (*p* > 0.05). The conversion rate to thoracotomy between TP-RATS and VATS showed a significant difference (0% vs. 17%, *p* = 0.041).

In the SP-RATS group, one patient required additional ports (two-port conversion) due to a narrow ICS. One patient required an unexpected conversion to VATS due to bleeding, but after a rapid control of the bleeding, the procedure was safely converted back to RATS and completed safely. In the TP-RATS group, three patients required an additional port (three-port conversion) due to severe pleural adhesions in two cases and abnormal anatomy in one case; two patients required conversion to VATS due to unexpected bleeding. There was no conversion to thoracotomy in either group. In the VATS group, seven patients required multi-port conversion due to severe pleural adhesions in three cases, difficult lymph node dissection in three cases, and abnormal anatomy in one case. Additionally, five patients required conversion to thoracotomy due to unexpected bleeding in four patients and difficult lymph node dissection in one case. There was no incidence of reoperation nor 30-day mortality.

Pathological findings are summarized in Table 4. There were no significant differences between the three groups in terms of histological type, number of lymph nodes harvested, and TNM staging (*p* > 0.05).

## 4. Discussion

To the best of our knowledge, this study is the first to report on SP-RATS lobectomy for patients with large-sized NSCLC. Thus, to establish its feasibility, we compared the perioperative outcomes of patients who underwent this approach with those of patients who underwent TP-RATS and VATS lobectomy. This study revealed that SP-RATS is not inferior to TP-RATS and VATS for large-sized NSCLC in terms of perioperative outcomes. Our findings suggest that the SP-RATS approach can be a valuable alternative surgical option for large-sized tumors, combining the potential benefits of robotic surgery and SP surgery.

Initially, an 8 cm utility incision at the seventh–eighth ICS level between the mid-axillary line and posterior axillary line was used; however, with increased experience, we successfully performed the SP-RATS approach using a 6 cm utility incision. If the wound incision is further narrowed, the risk of collision increases, and there is a higher possibility of specimen rupture during the removal of an overly large specimen. The Xi officially requires a distance of more than 8 cm between each robotic arm [20].

Gonzalez et al. first reported SP-RATS pulmonary resection using Xi in 2022 [15], and their approach featured a 3–4 cm incision at the sixth–seventh ICS level between the anterior axillary line and posterior axillary line without CO_2_ insufflation. Compared to their approach, our approach allows the assistant to more easily insert instruments due to the wider space between the robotic arms, with fewer collisions between the robotic arms, making it easier for the surgeon to initiate SP-RATS. Presently, SP-RATS is not widely used, and we believe the main reason is the steep learning curve for both surgeons and assistants. Our study highlights that with a larger incision, SP-RATS can be attempted more easily and effectively compared to traditional methods. Thus, CO_2_ insufflation can provide a superior surgical view. We usually utilize a CO_2_ insufflation pressure of 6 mmHg. Only in rare cases where the surgical view is not adequately obtained do we increase the CO_2_ pressure from 6 mmHg to 8–10 mmHg after confirming hemodynamic stability. Because we gradually increase the CO_2_ insufflation pressure, cases of cardiopulmonary problems are rare.

The efficacy of SP-RATS using the Xi remains controversial. Some surgeons have reported that this approach is feasible and safe, citing successful cases, while others have raised concerns about its safety and reproducibility. The current situation for SP-RATS is reminiscent of when VATS was first introduced, as well as when SP-VATS was initially presented. Sihoe et al. reported that for a new operative technique to become a standard treatment, it must go through five phases: (1) safety and feasibility; (2) crude benefit; (3) objective, quantifiable benefit; (4) treatment efficacy; and (5) sustainability [21]. Thirty years ago, many surgeons questioned the efficacy of VATS, but after going through the five phases, it became the mainstay for lung cancer surgery. However, SP-VATS did not overcome all five phases. SP-RATS has passed the first phase and is now approaching the second phase. For the global introduction and establishment of SP-RATS as a mainstay treatment, large-scale studies, including long-term outcomes, must provide clinical evidence, and an education system should be established to ensure reproducibility. The key difference between SP-VATS and SP-RATS is that the technological advancements in robotic systems are progressing rapidly and will continue to do so. This will likely increase the benefits of SP-RATS and make it easier for it to be more widely adopted.

Considering large-sized tumors, lung traction and suction during unexpected bleeding are particularly important, as is the ability to convert to VATS or thoracotomy, if necessary. Our approach allows the assistant to effectively perform lung traction and suction. An incision was made at the seventh–eighth ICS level to facilitate robotic stapler maneuverability, as the end effector of the robotic stapler is longer than that of other instruments. Although the incision site is at a lower level than typical incision sites for SP-VATS due to robotic stapling, our approach using a large utility incision facilitates conversion to VATS with rapid undocking. In this study, one patient required conversion to VATS due to unexpected bleeding; this was performed quickly and safely.

One concern with the SP-RATS approach for large-sized NSCLC is the prolonged total operative time. Several previous studies have demonstrated that RATS is associated with a longer total operative time than VATS [22,23,24,25,26]. In our SP-RATS approach, the total operative time may have been increased due to replacing the 8 mm port with the 12 mm port for stapling and then replacing the 12 mm port with an 8 mm port after stapling. To reduce replacing time, we used Hem-o-lok clips for the division of small vessels. Our results suggest no difference in the three groups in total operative time. The median total operative time (198 min) was comparable to that of conventional RATS lobectomy procedures recently reported in the literature (110–266 min) [12,27,28,29].

Our SP-RATS approach showed a postoperative complication rate of 36% (n = 4), with no major complications. These results for the total complication rate are similar to what is reported for patients with Stage II and III NSCLC (total complication rate of 14–46% and major complication rate of 3–13%) [7,30,31], although the major complication rate was low. Precise dissection using the robotic system may minimize postoperative complications. Specifically, the SP-RATS approach can facilitate meticulous dissection during severe adhesion better than that seen with multi-port RATS.

Several studies have shown the feasibility of RATS for locally advanced NSCLC [7,32,33]. To date, there have been no studies reporting the feasibility of RATS for large-sized NSCLC. Jiang et al. reported that RATS thymectomy for a large anterior mediastinal mass (≥6 cm) is safe and as effective as VATS and sternotomy [6]. For large-sized NSCLC, manipulating the lung to gain an optimal surgical view can be technically challenging, and there is a high risk of tearing small vessels, making the surgery dangerous. The robotic surgical platform facilitates complex surgeries, providing increased treatment options to patients who would have previously required thoracotomy. Although indications for RATS in patients with large-sized NSCLC have not yet been established, we believe that the tumor size itself is not an absolute contraindication for RATS.

The future of SP-RATS for large-sized tumors will be promising, along with the continued development of robotic technologies. The da Vinci single-port robotic surgical system (Intuitive Surgical Inc.) was developed to perform SP surgery. Notably, it has three articulating arms and one articulating robotic scope. We have previously reported the feasibility of this system for major pulmonary resection [16]. Although this system still has limitations, such as a large cannula size (2.8 cm) and the lack of a dedicated stapler, these issues are expected to improve in the future with technological advancements. Once these issues are resolved, SP-RATS using this system can be widely applied not only to large-sized tumors but also to patients with advanced-stage tumors. Therefore, future studies should include SP-RATS lobectomy using the SP robotic system for large-sized NSCLC.

This study has some limitations. First, it was a single-center retrospective study with a small sample size. During the study period, there were relatively few cases of RATS lobectomy for patients with large NSCLC because many patients with NSCLC > 5 cm underwent neoadjuvant chemotherapy to reduce tumor size before surgery or received concurrent chemoradiotherapy. Second, data on some variables, such as docking and undocking time, total time spent on the conversion process, and the amount of intraoperative bleeding, were not available due to the retrospective nature of the study. Third, long-term outcomes, including disease-free survival and 5-year survival, were not included in this study because, although oncologic outcomes are crucial for patients with NSCLC, many cases of SP-RATS are recent.

## 5. Conclusions

SP-RATS lobectomy for patients with large-sized NSCLC is feasible and safe. Our approach can be used as an alternative surgical option in selected cases and by experienced robotic surgeons. Although further large-sized studies are necessary to establish the clinical feasibility of this approach, the potential of SP-RATS for large-sized NSCLC is promising with advancements in robotic technologies.

## Figures and Tables

**Figure 2:**
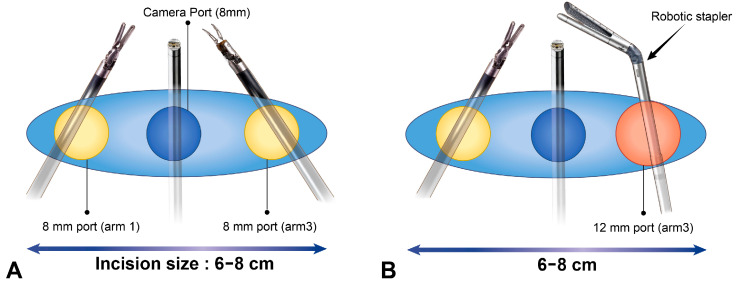
Port layout for stapling. (**A**) Usually, two 8 mm ports are placed at the left and right edges of the incision. (**B**) During stapling, an 8 mm port at the position where the stapler will be inserted is replaced with a 12 mm port.

**Table 1:** Detailed characteristics of patients who underwent single-port robotic-assisted thoracic surgery lobectomy.

Sex/Age (Years)	Tumor Size on CT/on Pathology (cm)	Diagnosis	Procedure	Incision Size	Chest Tube Duration/ Postoperative LOS (Days)	Intraoperative/Postoperative Complications
M/48	9.1/7.6	Sqcc	LLL lobectomy	8	2/4	None
M/69	7/6.5	Sqcc	LUL lobectomy	7	3/4	None
F/52	5.5/5.1	Other	RML + RLL bilobectomy	8	2/4	None
M/52	7/0 (ypT0)	Adenoca	LUL lobectomy	8	2/3	None
M/50	6/5.1	Adenoca	RUL lobectomy	8	2/4	None
M/73	7.5/7.1	Adenoca	RLL lobectomy	7	3/4	None
M/60	6.6/6.1	Adenoca	LUL lobectomy	7	6/8	Conversion to VATS/Arrhythmia, Persistent air leak
M/87	6.1/6	Sqcc	LLL lobectomy	6	5/15	Conversion to Two-port/Pneumonia
F/73	7.5/7/2	Adenoca	RLL lobectomy + RUL wedge	6	2/4	None
F/70	6.5/6	Sqcc	RML + RLL bilobectomy	7	9/10	Persistent air leak
F/66	6.4/5.6	Adenoca	RLL lobectomy + RUL wedge	6	5/12	Pneumonia

CT, computed tomography; Sqcc, squamous cell carcinoma; Adenoca, adenocarcinoma; LOS, length of stay; VATS, video-assisted thoracic surgery.

**Table 2:** Patient characteristics.

Variables	SP-RATS	TP-RATS	VATS	*p*-Value		
	(n = 11)	(n = 24)	(n = 29)	SP-RATS vs. TP-RATS	SP-RATS vs. VATS	TP-RATS vs. VATS
Age (years)	66 [52–73]	66 [61–74]	69 [65–76]	0.723	0.110	0.105
Sex, male	7 (64%)	13 (54%)	22 (76%)	0.721	0.455	0.097
BMI (kg/m^2^)	24.39 [21.30–27.67]	24.17 [20.52–26.55]	24.29 [21.50–26.18]	0.715	0.647	0.952
Pulmonary function						
FEV_1_ (%)	83 [76–90]	85 [74–92]	82 [76–88]	0.965	0.681	0.543
DLCO (%)	80 [72–91]	80 [73–90]	75 [71–88]	0.746	0.737	0.260
Comorbidities, n (%)						
HTN	5 (45%)	11 (46%)	15 (52%)	0.983	0.723	0.669
DM	3 (27%)	9 (37%)	6 (21%)	0.709	0.686	0.176
TB	1 (9%)	2 (8%)	4 (14%)	1.000	1.000	0.532
COPD	0	1 (4%)	3 (10%)	1.000	0.548	0.617
Smoking status				0.458	0.898	0.424
Never	4 (36%)	14 (58%)	13 (45%)			
Ex	5 (45%)	8 (33%)	10 (34%)			
Current	2 (18%)	2 (8%)	6 (21%)			
Alcohol use				1.000	1.000	0.530
No	6 (54%)	12 (50%)	17 (59%)			
Yes	5 (45%)	12 (50%)	12 (41%)			
ASA score	3 [2–3]	3 [2–3]	3 [2–3]	0.137	0.611	0.228
Tumor size on CT (cm)	6.6 [6.1–7.5]	6.2 [6–7]	6.2 [5–7.5]	0.212	0.187	0.375
Tumor size on pathology (cm)	6 [5.1–7.1]	5.5 [5.1–6]	6 [5.3–7.7]	0.206	0.659	0.063
Tumor location				0.683	0.378	0.105
RUL	1 (9%)	4 (17%)	9 (31%)			
RML	0	1 (4%)	0			
RLL	4 (32%)	13 (54%)	8 (28%)			
LUL	3 (24%)	4 (17%)	4 (14%)			
LLL	2 (16%)	2 (8%)	8 (28%)			

SP-RATS, single-port robotic-assisted thoracic surgery; TP-RATS, two-port robotic-assisted thoracic surgery; VATS, video-assisted thoracic surgery; BMI, body mass index; FEV_1_, forced expiratory volume in 1 s; DLCO, diffusing capacity for carbon monoxide; HTN, hypertension; DM, diabetes mellitus; TB, tuberculosis; COPD, chronic obstructive pulmonary disease; CT, computed tomography. Data are expressed as number (%) and median [interquartile range].

**Table 3:** Perioperative outcomes.

Variables	SP-RATS	TP-RATS	VATS	*p*-Value		
	(n = 11)	(n = 24)	(n = 29)	SP-RATS vs. TP-RATS	SP-RATS vs. VATS	TP-RATS vs. VATS
Total operative time (min)	198 [159–260]	181.5 [165.5–212.5]	178 [154–232]	0.817	0.496	0.706
R0 resection	11 (100%)	23 (96%)	26 (90%)	1.000	0.548	0.447
Type of surgery				0.803	0.590	0.617
Lobectomy	7 (63%)	16 (67%)	22 (76%)			
Lobectomy + wedge	2 (18%)	2 (8%)	3 (10%)			
Lobectomy + segmentectomy	0	0	1 (3%)			
Sleeve lobectomy	0	2 (8%)	1 (3%)			
Bilobectomy	2 (18%)	4 (17%)	1 (3%)			
Pneumonectomy	0	0	1 (3%)			
Conversion						
Additional port	1 (9%)	3 (12%)	7 (24%)	1.000	0.405	0.318
VATS	1 (9%)	2 (8%)		1.000		
Open	0	0	5 (17%)		0.298	0.041
Chest tube duration (days)	3 [2–5]	3 [3–4.75]	3 [2–5]	0.532	0.767	0.845
Postoperative LOS (days)	4 [4–10]	5 [4.25–8.5]	6 [5–10]	0.494	0.265	0.498
Postoperative pain (VAS score)						
POD 0 pain	4 [3–5]	4 [3–5.75]	4 [3–7]	0.563	0.538	0.697
POD 1 pain	3 [2–3]	3 [2–3]	3 [2–5]	0.938	0.186	0.071
POD 2 pain	2 [2–3]	2 [2–3]	3 [2–3]	0.640	0.398	0.669
Postoperative complications				1.000	0.723	0.057
None	7 (63%)	15 (62%)	15 (52%)			
Minor complication (Grade I–II)	4 (36%)	6 (25%)	14 (48%)			
Major complication (Grade III–V)	0	3 (12%)	0			

SP-RATS, single-port robotic-assisted thoracic surgery; TP-RATS, two-port robotic-assisted thoracic surgery; VATS, video-assisted thoracic surgery; LOS, length of stay; VAS, visual analog scale; POD, postoperative day. Data are expressed as number (%) and median [interquartile range].

**Table 4:** Patient pathology.

Variables	SP-RATS	TP-RATS	VATS	*p*-Value		
	(n = 11)	(n = 24)	(n = 29)	SP-RATS vs. TP-RATS	SP-RATS vs. VATS	MP-RATS vs. VATS
Histological type				0.489	1.00	0.409
Adenoca	6 (55%)	17 (71%)	16 (55%)			
Sqcc	4 (36%)	6 (25%)	9 (31%)			
Other	1 (9%)	1 (4%)	4 (14%)			
Number of lymph nodes harvested	24 [19–26]	18 [14.25–24]	20 [10.5–26]	0.194	0.154	0.759
TNM staging				0.596	0.829	0.089
ypT0N0	1 (9%)	0	0			
ypT1cN0	0	1 (4%)	0			
ypT2aN0	0	0	1 (3%)			
ypT2aN1	0	0	1 (3%)			
ypT4N0	0	0	1 (3%)			
T2bN0	0	2 (8%)	3 (10%)			
T2bN1	0	0	1 (3%)			
T3N0	3 (27%)	8 (33%)	8 (27%)			
T3N1	2 (18%)	4 (17%)	2 (7%)			
T3N2	2 (18%)	7 (29%)	2 (7%)			
T4N0	2 (18%)	1 (4%)	7 (24%)			
T4N1	1 (9%)	1 (4%)	2 (7%)			
T4N2	0	0	1 (3%)			

SP-RATS, single-port robotic-assisted thoracic surgery; TP-RATS, two-port robotic-assisted thoracic surgery; VATS, video-assisted thoracic surgery; Adenoca, adenocarcinoma; Sqcc, squamous cell carcinoma. Data are expressed as number (%) and median [interquartile range].

## Data Availability

The data underlying this article will be shared upon reasonable request to the corresponding author.

## Supplementary Materials

The following supporting information can be downloaded at https://www.mdpi.com/article/10.3390/cancers16173091/s1, Video S1: Movement of robotic arms in the single-port robotic-assisted thoracic surgery approach using the da Vinci Xi robotic system, Video S2: The surgical video of single-port robotic-assisted thoracic surgery left lower lobe lobectomy for large-sized non-small cell lung cancer.

## Abbreviations

Adenoca	adenocarcinoma
BMI	body mass index
COPD	chronic obstructive pulmonary disease
CO_2_	carbon dioxide
CT	computed tomography
DLCO	diffusing capacity for carbon monoxide
DM	diabetes mellitus
FEV_1_	forced expiratory volume in 1 s
HTN	hypertension
ICS	intercostal space
IQR	interquartile range
LOS	length of stay
NSCLC	non-small cell lung cancer
RATS	robotic-assisted thoracic surgery
SP	single-port
SP-RATS	single-port robotic-assisted thoracic surgery
SP-VATS	single-port video-assisted thoracic surgery
Sqcc	squamous cell carcinoma
TB	tuberculosis
TP-RATS	two-port robotic-assisted thoracic surgery
Xi	da Vinci Xi robotic surgical system
